# Post-dural Puncture Headache: A Comparative Study Using 25 G Quincke’s Needle in Midline and Paramedian Approaches in Patients Undergoing Elective Cesarean Section

**DOI:** 10.7759/cureus.66656

**Published:** 2024-08-11

**Authors:** Dnyanshree Wanjari, Nikhil Bhalerao, Amreesh Paul, Amol Bele

**Affiliations:** 1 Anaesthesiology, Jawaharlal Nehru Medical College, Datta Meghe Institute of Higher Education and Research, Wardha, IND; 2 Anaesthesiology, Imambara District Hospital, Hooghly, IND

**Keywords:** pregnant females, central neuraxial blockade, spinal anaesthesia, post-dural puncture headache, paramedian approach, midline approach

## Abstract

Background and objectives

Spinal anesthesia (SA) has become a preferred anesthetic technique for elective cesarean sections due to its rapid onset, profound sensory and motor blockade, and minimal impact on the newborn. It lowers the risk of development of thrombus in the veins and pulmonary vessels and permits early ambulation. The most popular technique used to reach the subarachnoid space is the midline technique, though it can be challenging to use in some cases, including those involving elderly patients with degenerative abnormalities in the vertebral column, patients who are unable to flex the vertebral column, noncooperative patients, and hyperesthetic patients. The paramedian technique resolves the challenges posed by the midline technique. It is also relatively easy to carry out. Based on the midline technique's inadequacies, we hypothesized that the paramedian method of SA would be less complicated than the midline approach, with a relatively low occurrence of post-dural puncture headaches (PDPH).

Methodology

Using the midline and paramedian approaches during cesarean surgeries, we performed an observational descriptive longitudinal study to assess the occurrence and magnitude of PDPH. During an elective cesarean delivery, the seated patient received 2.0-2.5 ml of hyperbaric bupivacaine using the midline or paramedian approaches and a 25 G Quincke's needle at the L3-L4 level. Eighty-four pregnant females with American Society of Anesthesiologists (ASA) physical status II, aged 18 to 35 (n = 42 in each group), were included in this research. The occurrence and severity of PDPH were compared among the groups during a period of five days.

Result

In comparison to the paramedian group (7.1%), the midline group had a higher incidence of PDPH (14.3%). There was a significant correlation between the technique and the occurrence of PDPH (p = 0.041). The visual analogue scale (VAS) was employed to quantify pain five days after surgery. Pain levels in Group B (paramedian) were consistently less than those in Group A (midline). On day 1, Group B had a mean score of 0.49 ± 1.16 (p = 0.030) compared to Group A's mean VAS score of 1.27 ± 1.95. Day 5 (p = 0.032): Because this tendency persisted through day 5, the p-values for days 2, 3, 4, and 5 remained significant. These findings suggest that the midline technique is linked to a higher occurrence and magnitude of PDPH than the paramedian approach.

Conclusion

Employing a paramedian technique has been associated with a noteworthy decline in the frequency of PDPH and a decrease in the need for additional analgesics, which could lead to a less severe case of PDPH. The paramedian approach needed fewer attempts and needle passes, which leads to a lower incidence of headache, backache, and injection site pain and better patient satisfaction.

## Introduction

In 1898, August Karl Gustav Bier requested spinal anesthesia (SA) from a colleague, which resulted in him experiencing excruciating headaches. Most frequently, spinal or accidental dural punctures following an epidural space-related diagnostic or therapeutic procedures result in post-dural puncture headaches (PDPHs) [1]. PDPH is a headache that occurs seven days following a spinal puncture, occurs or worsens within 15 minutes of attaining a sitting position, and improves within 30 minutes of lying supine [2]. During SA or subarachnoid block, a form of central neuraxial blockade, a local anesthesia drug is administered into the subarachnoid area [3]. The paramedian technique and the midline procedure are utilized to make a subarachnoid space entry. SA lowers the risk of the development of deep vein thrombosis and permits early ambulation. With SA, a woman undergoing a cesarean section can remain alert and awake, allowing her to experience her child's birth and early rooming [4].

PDPHs are among the most frequent and adverse after-effects of SA. Several circumstances, including pregnancy, increase the risk of PDPH. The obstetric age range, which is also the period with the highest incidence of PDPH, is 18 to 35. The frequency of PDPH decreases in younger individuals (under 13) and older adults (over 60). A high incidence of PDPH is associated with pregnancy-related raised cerebrospinal fluid (CSF) pressure, blood loss, postpartum diuresis, hormonal imbalance, and elevated serum estrogen level in obstetric patients [5]. Several propositions have been suggested to explain the development of PDPH. It is undeniable that CSF loss lowers CSF pressure, even if it is unclear exactly which mechanism causes the headache. Bier first proposed the hypothesis of continuous leakage as the cause of pain, which developed into Kunkle's 1943 theory of traction. This idea suggests that decreased CSF pressure causes sagging of the brain and meninges, generating traction in areas sensitive to pain. The vascular theory is an alternate idea that states that the loss of CSF immediately activates adenosine receptors. This causes the venous sinuses and cerebral veins to dilate compensatorily, which in turn causes the pain-sensitive fibers in the cerebrum to stretch [6]. Still, the frequency of occurrence of PDPH with SA is not very clear. The most widely used technique for SA is the midline approach. Still, technically, it is a bit difficult, more so for older people with structural changes in the spine resulting from degeneration. The landmarks of spinal interspace are compromised in obese and edematous patients. This technique can be done in obese, elderly, and pregnant patients because the paramedian technique makes it easy to simplify the location of the needle insertion and makes it possible to do the procedure in an unflexed spinal position [4]. 

With the use of a 25 G Quincke's needle for SA administered via the paramedian and midline procedures, this study sought to evaluate the incidence and severity of PDPH in patients who had undergone a cesarean section. The primary objective of this study was to compare the incidence and severity of PDPH in patients who have gone through elective cesarian surgery using the midline and paramedian approaches. Secondary objectives were assessing the number of spinal needle insertion attempts and needle passes required, comparing the incidence of backache, documenting the occurrence of paresthesia during needle insertion, and comparing the incidence of nausea and vomiting in patients receiving SA through both approaches. 

## Materials and methods

This was an observational descriptive longitudinal study conducted for two years in the obstetrics and gynecology operating room and ward in the Imambara District Hospital in Hooghly, West Bengal, after approval from the institutional scientific review board (approval number: RKC SS/Review/257). All patients aged between 18 and 35 years attending the Obstetrics and Gynecology Department of Imambara District Hospital, Hooghly, West Bengal, scheduled for elective cesarean sections formed the sample. The inclusion criteria for this study were informed consent from patients, only ASA grade II, pregnant women between 18 and 35 years old, and those booked for elective cesarean section by SA. Patients who will be excluded from this are those who refuse; have psychological disorders, prior spinal cord surgery; ASA grades III and IV; spinal anatomical deformities; coagulopathies; systemic toxicity history, either from the local anesthetics to be used or any other local anesthetic medications.

Sample size

The incidence of PDPH in 120 expectant mothers who had elective cesarean sections was compared between the two techniques by Behary and Elshat [7]. Of these patients, 56 had the midline approach, and 58 had the paramedian approach. According to their findings, patients who received the paramedian technique had a much-decreased incidence of PDPH. The paramedian technique was also linked to a decreased incidence of paresthesia and backache. The following formula was utilized to determine the study's sample size: N = (p1 − p2)^2^[Z(1 − α/2)P(1 − P)+Z(1 − β)p1(1 − p1)+p2(1 − p2)]^2^.

In this computation, the sample size is represented by N. The values of Z(1 − α/2) and Z(1 − β) are 1.96 and 0.84, respectively, for 𝑍(1 − 𝛼 / 2) and Z(1 − α/2), respectively. The average of P1 and P2 is P, where P1 is 19.6% and P2 is 5.2%. This formula gives 42 as the estimated sample size.

Methodology

All consenting patients were scheduled for elective cesarean sections under SA. From the patient pool, 42 patients were identified for Group A (midline technique) and 42 patients for Group B (paramedian technique). Patients were randomized to one of two groups to undergo SA via the midline (Group A) or paramedian (Group B) techniques using random number generator software. Spinal anesthesia was provided to each group by a senior consultant anesthesiologist who has performed over 100 SA procedures. The patient's transfer to the operating room followed the pre-anesthesia evaluation. Following ASA standards, a noninvasive monitor was connected as soon as the patients were brought to the operation room, and intravenous access was established using a 16/18G venous cannula. The patients in both groups were preloaded with 500 ml of Ringer lactate. No premedication was given. The patients were then made to sit on a leveled trolley with footrests supporting their feet. They were told to keep their necks flexed and backs arched, and the patient was put in a position that would sustain the position while an aide was holding the patient. They were also provided pillows to hug. Before the procedure, the anesthesiologist scrubbed in a sterile gown, gloves, cap, and mask. The anesthesiologists palpated for landmarks after applying 2% chlorhexidine to the skin. Following confirmation of the chosen intervertebral space, 2 ml to 5 ml of 1% lidocaine was injected into the skin.

**Figure 1:**
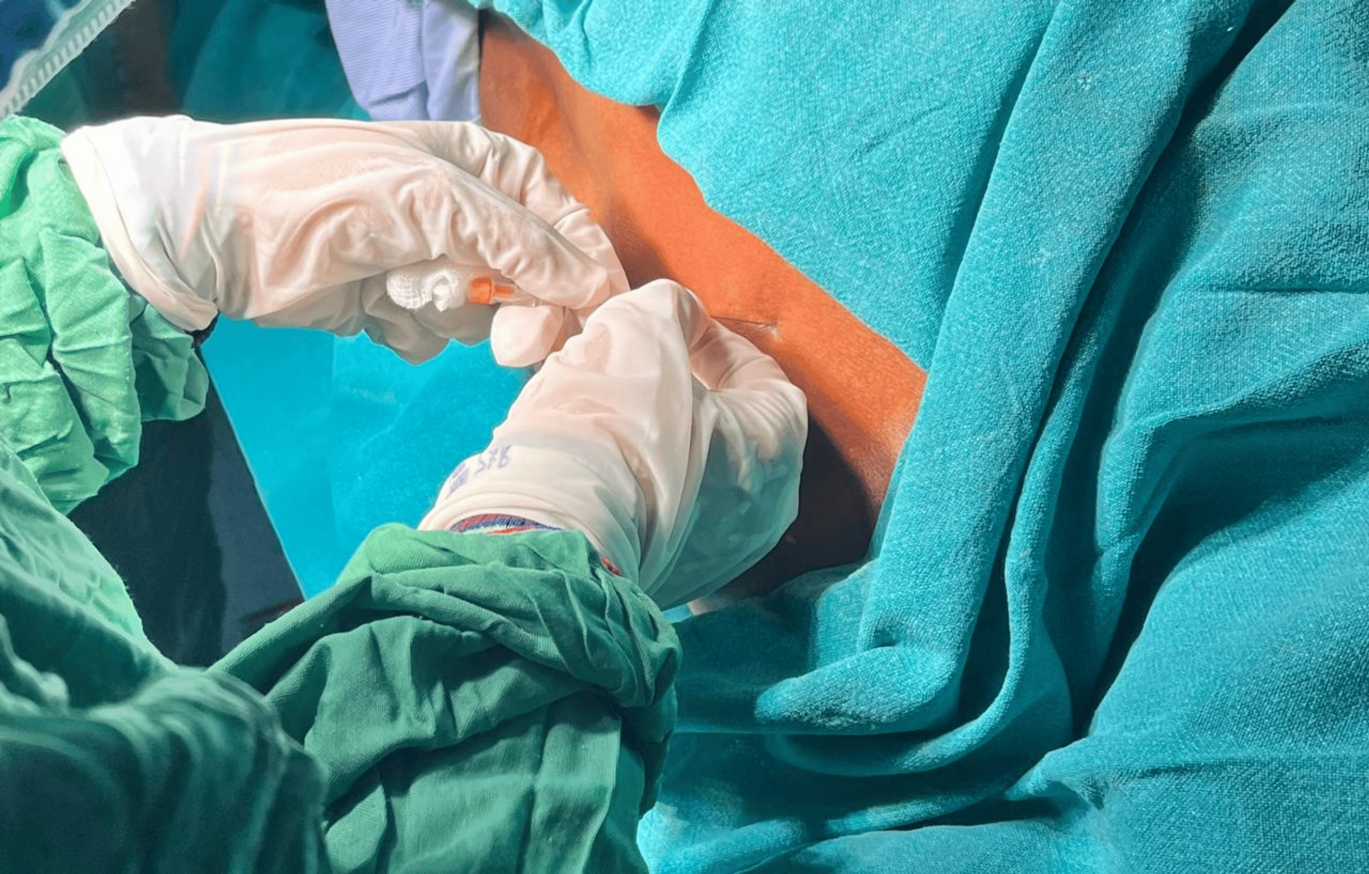
Spinal anesthesia administered using a 25 G Quincke's needle by the midline technique

While patients in Group B underwent SA using the paramedian approach, those in Group A underwent SA using the midline technique (Figure 1). A free flow of CSF was obtained by puncturing the dura using a 25 G Quincke's needle, and 2.0-2.5 ml of 0.5% hyperbaric bupivacaine was administered. Following SA, patients were suitably positioned and made to lie supine. Patients in both groups had noninvasive blood pressure, heart rate, and pulse oximetry monitoring during the surgical operation. Following surgery, patients were taken to a ward and kept under strict observation for five days. Monitoring was done on the following patient outcomes: headache, backache, paresthesia, nausea, vomiting, pain at the injection site, necessity of additional analgesics, and patient satisfaction score following a spinal puncture. The number of needle passes and attempts was also recorded. The intensity of the headache was assessed using the VAS. Patients received intravenous injections of 50 mg of tramadol and 1 g of paracetamol to relieve PDPH. Number of spinal needle insertion attempts, the needle passes, the incidence of PDPH, VAS scores for PDPH over five postoperative days, the incidence of backache, the occurrence of paresthesia, the incidence of nausea and vomiting, pain at the injection site, the need for additional analgesics, and patient satisfaction score were among the significant factors that the observer observed.

Definition of parameters

The entire number of forward spinal needle advancements in one interspinous site, including the needle's withdrawal and redirection without removing it from the skin, is known as the number of passes. The total number of spinal needle insertion attempts is the total number of times the needle is taken out and put back into the patient's skin. Patients were asked to verbally score their level of discomfort on an 11-point VAS scale following surgery, where 0 represented no pain and 10 the worst possible suffering. Postoperative VAS pain scores were recorded for PDPH for a maximum of five days following surgery. Patient satisfaction score was evaluated using Likert scale from 0 to 5. 

Statistical methods

To help with the statistical analysis, the Centers for Disease Control and Prevention (CDC) trademark Epi Info (TM) 3.5.3 was employed. Cross-tabulations and basic frequency distributions were produced using this software. The corrected test was used if it was found that one of the cell frequencies in the bivariate frequency distribution was less than five. The Z-test, also known as the test of proportion, was used to determine the significance of the difference between two proportions. The mean deviation of the significance was checked through a t-test. A p-value less than 0.05 was considered statistically significant.

## Results

Patients in Group A had a mean age of 24.74 ± 3.83 years; in Group B, patients had a mean age of 24.79 ± 3.79 years (p = 0.95; t82 = 0.057, NS). There is no statistically significant age difference between the two groups; implying that the ages are well-matched. The mean body mass index (BMI) in Group A was 24.61 ± 3.93 kg/m² and in Group B was 26.19 ± 3.55 kg/m² (t82 = 1.126; p = 0.44, NS). This means that there was no significant difference in BMI. The mean number of tries in Group A was 1.76 ± 0.43, significantly greater than the mean number of attempts in Group B, 1.07 ± 0.26. This indicates a statistically significant difference. It took more tries for Group A to achieve effective anesthesia. With t82 = 3.40 and p < 0.001*, Group A's average spinal needle insertion attempts were 2.80 ± 0.98, considerably greater than Group B's average of 1.31 ± 0.60. As a result, there is a substantial difference in the total number of needle passes between the two groups, with Group A having more spinal needle insertion attempts. These are illustrated in Table 1.

**Table 1:** Comparison of parameters between the two groups of patients Group A: Midline technique; Group B: paramedian technique

	Group A	Group B	t-test value	p-value
Age (in years)	24.74 ± 3.83	24.79 ± 3.79	0.057	0.95
BMI (in kg/m^2^)	24.61 ± 3.93	26.19 ± 3.55	1.126	0.44
Number of attempts	1.76 ± 0.43	1.07 ± 0.26	2.52	0.005
Number of spinal needle insertion attempts	2.80 ± 0.98	1.31 ± 0.60	3.40	<0.001*

Nine patients in Group A and three in Group B had PDPH (p = 0.041). As can be seen in Table 2, the result is statistically significant since it indicates that Group A had a larger incidence of PDPH than Group B.

**Table 2:** Comparison of occurrence of PDPH in the patients of the two groups Group A: Midline technique; Group B: paramedian technique

Occurrence of PDPH	Group A	Group B	Total
Yes	9	3	12
No	33	39	72

Group A exhibited a greater mean pain score than Group B from days 1 to 5 following surgery (p < 0.01 over all periods). This identifies a statistically significant difference between the two groups' pain levels; Group A's pain levels are higher, as shown in Table 3.

**Table 3:** Comparison of severity of pain according to VAS at different time intervals of the patients of the two groups VAS: Visual analogue scale; Group A: midline technique; Group B: paramedian technique

	Group A	Group B	t- test value	p-value
VAS on day 1	1.27 ± 1.95	0.49 ± 1.16	2.214	0.030*
VAS on day 2	1.51 ± 2.06	0.53 ± 1.10	2.691	0.009*
VAS on day 3	1.61 ± 2.29	0.47 ± 1.14	2.878	0.006*
VAS on day 4	0.93 ± 1.66	0.12 ± 0.39	3.040	0.004*
VAS on day 5	0.39 ± 0.97	0.05 ± 0.21	2.215	0.032*

The list of complications appears in Table 4. Backaches were reported by two patients (4.8%) and six patients (14.3%) from Group B and Group A, respectively (χ² = 3.61; p = 0.044, S). It can be concluded that there is a statistically significant difference between Group A and Group B in terms of backache. Two (4.8%) and three (7.1%) of Group B's members reported experiencing paresthesia ( NS, χ² = 0.21, p = 0.64). Therefore, the incidence of paresthesia in the two patient groups does not differ statistically significantly. The frequency of nausea and vomiting did not significantly differ between the two groups; three patients in Group B and four in Group A (9.5% and 9.5%, respectively) reported similar symptoms (χ² = 0.15; p = 0.69). Pain at the injection site was reported by two patients (4.8%) in Group B and eight patients (19.0%) in Group A (χ² = 2.54; p = 0.11, NS). This suggests that there is no appreciable variation in the two groups' injection site pain sensations.

**Table 4:** Comparison of the occurrence of complications between the two groups Group A: Midline technique; Group B: paramedian technique

	Group A	Group B	p-value
Backache	6	2	0.044
Paresthesia	3	2	0.64
Nausea and vomiting	4	3	0.69
Pain at injection site	8	2	0.11

Six patients (14.3%) in Group A required analgesic drugs, while one patient (2.4%) from Group B did (χ² = 4.08; p = 0.043, S). The conclusion could be that there is a statistically significant difference. Group A had a greater requirement for analgesic drugs than Group B (Table 5).

**Table 5:** Requirement of rescue analgesics in both groups Group A: Midline technique; Group B: paramedian technique

Need of analgesic drugs	Group A	Group B	Total
Yes	6	1	7
No	36	41	77

The satisfaction score was calculated using the Likert scale from 0 to 5. The score in patients from Group A was 3.34 ± 0.76 and that for Group B was 2.28 ± 0.67. The t₈₂ value is -1.125 with p = 0.264. This means that there were no statistically significant differences in mean satisfaction scores between the two groups of patients, thus showing comparable levels of satisfaction among the patients (Table 6).

**Table 6:** Patient satisfaction score Group A: Midline technique; Group B: paramedian technique

	Group A	Group B	t-test value	p-value
Patient satisfaction score	3.34 ± 0.76	2.28 ± 0.67	1.125	0.264 NS

## Discussion

**Figure 2:**
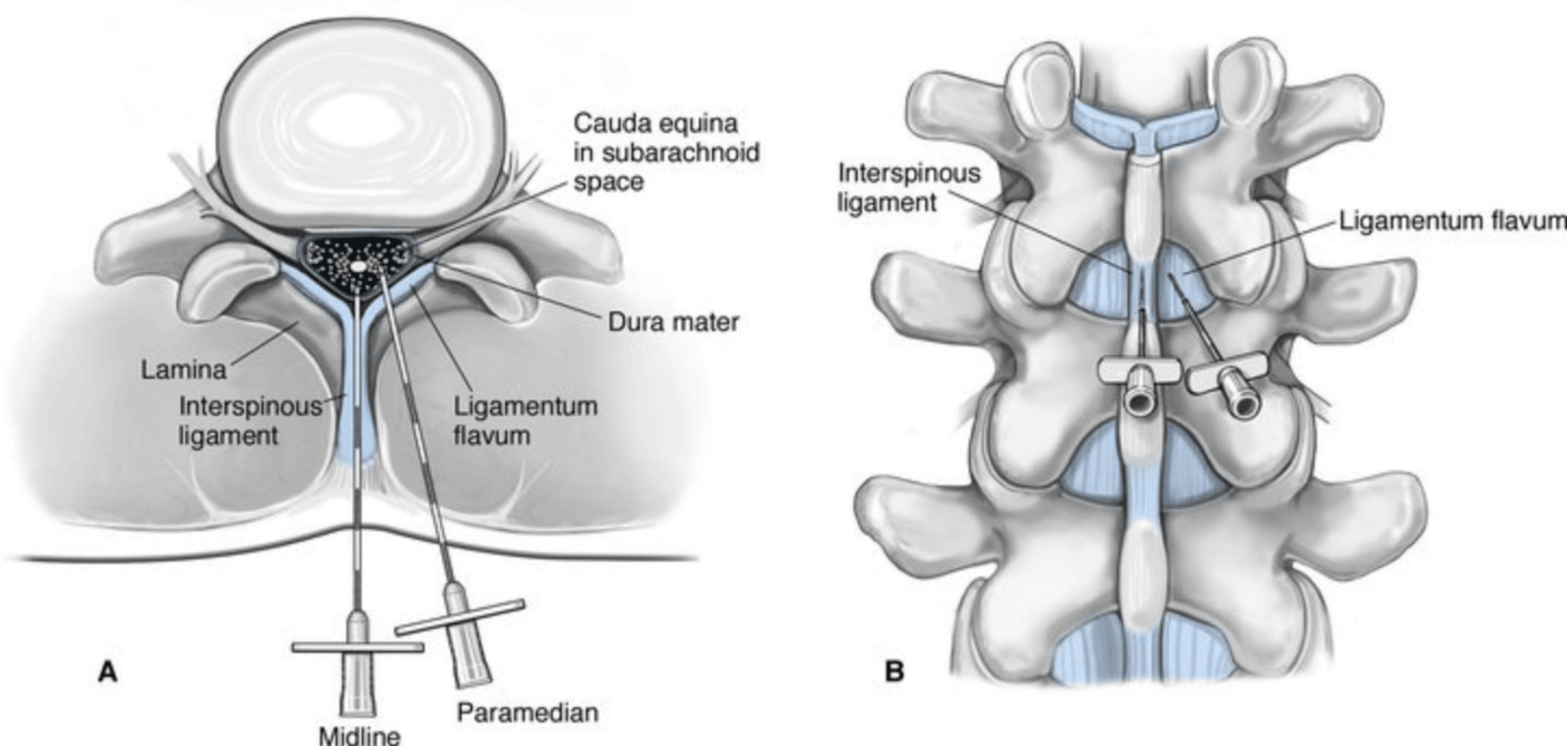
Midline vs paramedian approach to spinal anesthesia Image taken from https://www.cambridge.org/core/books/abs/essential-clinical-anesthesia/spinalanesthesia/A16A51E271FC06DF7D2AC6942B03F5C9

SA may be administered for lower abdominal surgeries, transurethral surgeries, orthopedic surgeries, and cesarean sections. The midline and paramedian approaches are used to provide subarachnoid block. Advantages of the paramedian approach over the classical midline approach to SA therefore exist by avoiding interspinous and supraspinous ligaments, which can be difficult to negotiate in patients with calcified ligaments or distorted anatomy. The approach has thus been found useful, particularly in elderly patients and those with degenerative changes whose anatomy may be distorted or the midline structures less clear and more rigid. By missing the midline ligaments, the trajectory of the paramedian approach has less resistance from dense ligamentous tissue and therefore less resistance to the insertion of the needle (Figure 2). Moreover, it minimizes the risk of vertebral bone contact, especially in those patients who have scoliosis or spinal deformities [4].

One of the most typical adverse effects of SA is headaches after a spinal puncture. The reason for it is a decrease in CSF volume and pressure brought on by the dural puncture which causes a loss of CSF from the intrathecal area. Several elements, such as age, sexual orientation, BMI, spinal needle type and size, needle bevel direction, and technique, might result in PDPH [8].

In one study, Rabinowitz et al. analyzed the success rates of the two approaches in 40 patients [9]. They demonstrated the superiority of the paramedian approach, with an 85% success rate compared to a 45% success rate for the more conventional midline approach. A total of 84 patients undergoing lower segment cesarean sections as elective procedures were assessed in this study. Group A and Group B were given anesthesia using the midline and paramedian approaches, respectively. All demographic parameters, such as age and BMI, were statistically insignificant between the groups. Previous studies by Wadud et al. showed a variation in the PDPH frequency depending on the patient's age: the older the patient, the less frequent the PDPH [10]. That was supported by recent research done by Peralta et al. and Miu et al. on the dependence of BMI on PDPH incidence, which showed no statistically significant variations in BMI between the two groups [11,12].

On procedural efficiency, Group A required more attempts and more passes of the needle than Group B. This goes on to prove that a paramedian approach is easier to perform with better success rates as compared to others, which other studies by Seeberger et al. and Khraise et al. have supported [13,14]. Now, the incidence of PDPH in Group B was significantly lower at 7.1% as compared to the midline group at 14.3%, and this finding is further substantiated by prior studies from Haider et al., Janik et al., and Bansal et al. [15-17]. In addition, Group B's pain VAS levels were significantly lower when matched against those with Group A's at all times, which indicated that the PDPH in the midline approach group was more severe.

Group B's lower number of patients requiring additional analgesics for PDPH management further supports the method's effectiveness in lowering the condition, comparable to the researches by Mosaffa et al. and Uppal et al. [18,19]. Although a trend toward higher patient satisfaction scores in Group A was seen, these did not achieve statistical significance; overall, the patient experience seemed to be similar with both approaches, in accordance with the study done by Harrison et al. [20].

Limitations

First, the possible generalizations of findings will also be limited because they are performed at only one center. Second, the subjective nature of the observations within the study incorporates variability into the results, especially concerning the intensity of pain in different patients under the same conditions. With a follow-up period of five days postoperatively, this study will likely miss any PDPHs, which continue over a prolonged period. Moreover, 84 patients in one study is a small sample size. Thus, it cannot represent the broad geographical and demographic variations; hence, more studies on bigger and more diverse patient populations must be considered. Finally, the study strictly examined conventional midline or paramedian approaches for spinal anesthesia and could not further elucidate possible ways ultrasound-guided techniques may impact outcomes. Moreover, 25 G Quincke's needles in the study exclude examining the influence of needle size and type.

## Conclusions

Based on our research, we conclude that the paramedian method of spinal anesthesia has distinct benefits over the midline method in terms of lowering the incidence and severity of PDPH. The study demonstrates that the paramedian technique for spinal anesthesia in cesarean surgeries significantly reduces the incidence and severity of PDPH compared to the midline approach. The paramedian method is associated with fewer attempts and needle passes, resulting in a lower incidence of headache, backache, and injection site pain, contributing to improved patient satisfaction. 

## Figures and Tables

**Figure 3:**
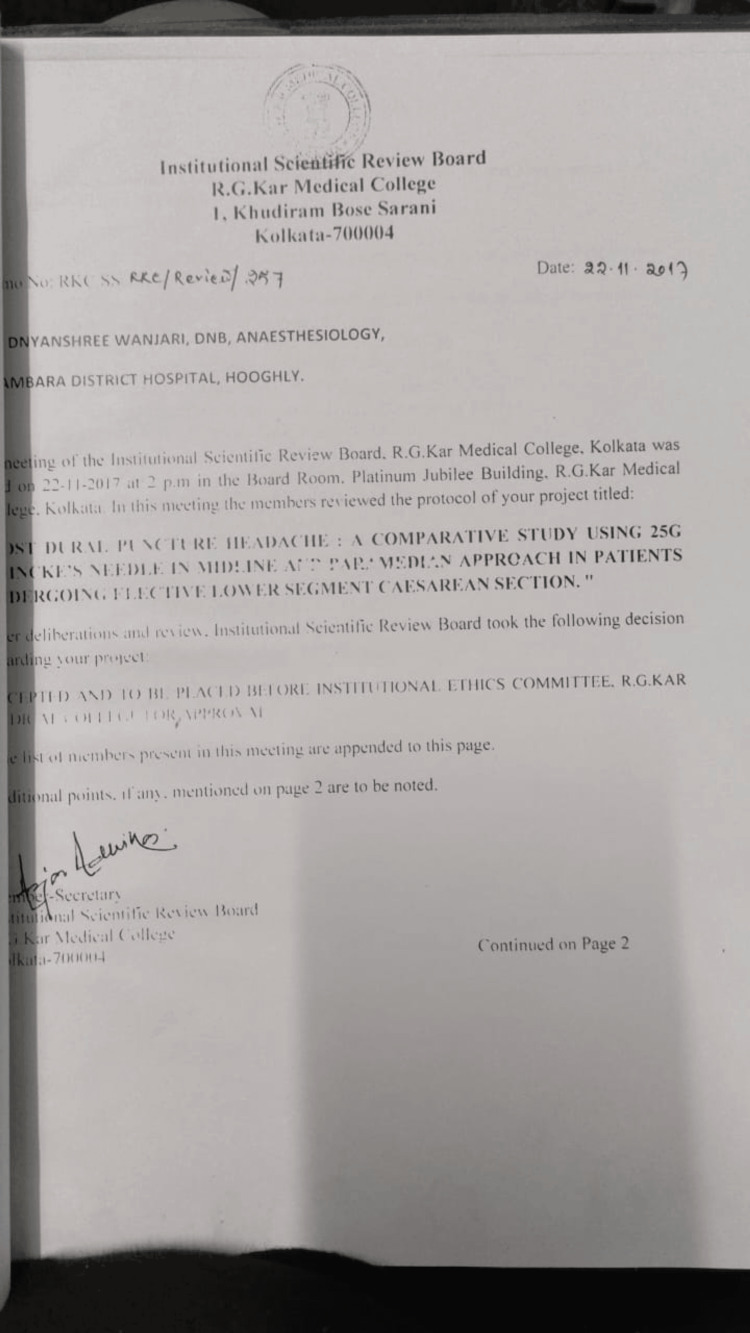
Ethic approval statement
